# A Surface Plasmon Resonance-based assay to measure serum concentrations of therapeutic antibodies and anti-drug antibodies

**DOI:** 10.1038/s41598-018-37950-4

**Published:** 2019-02-14

**Authors:** Marten Beeg, Alessandro Nobili, Barbara Orsini, Francesca Rogai, Daniela Gilardi, Gionata Fiorino, Silvio Danese, Mario Salmona, Silvio Garattini, Marco Gobbi

**Affiliations:** 1 Istituto di Ricerche Farmacologiche Mario Negri IRCCS, Milan, Italy; 2 Clinical Gastroenterology Unit, Careggi University Hospital, Florence, Italy; 3 IBD Center, Dept. of Gastroenterology, Humanitas Clinical and Research Institute, Rozzano, Milan, Italy; 4 Department of Biomedical Sciences, Humanitas University, Rozzano, Milan, Italy

## Abstract

Therapeutic drug and immunogenicity monitoring (TDIM) is increasingly proposed to guide therapy with biologics, characterised by high inter-individual variability of their blood levels, to permit objective decisions for the management of non-responders and reduce unnecessary interventions with these expensive treatments. However, TDIM has not yet entered clinical practice partly because of uncertainties regarding the accuracy and precision of enzyme-linked immunosorbent assays (ELISA). Here we report the characterisation of a novel surface plasmon resonance (SPR)-based TDIM, applied to the measurement of serum concentrations of infliximab, an antibody against tumour necrosis factor α (anti-TNFα), and anti-infliximab antibodies. SPR has the obvious advantages of directly detecting and measuring serum antibodies in minutes, avoiding the long incubation/separation/washing/detection steps of the methods proposed so far, reducing complexity and variability. Moreover, drug and anti-drug antibodies can be measured simultaneously. This new method was validated for sensitivity and reproducibility, and showed cost-effectiveness over commercial ELISA kits. This method may be applied to other biotherapeutics. These data pave the way for the development of SPR-based point-of-care devices for rapid on-site analysis.

## Introduction

Therapeutic antibodies are one of the most innovative and fastest growing segments in the pharmaceutical industry^1^, promoted by the continuous progress of molecular engineering technologies^2^. In comparison with conventional small-molecule drugs, monoclonal antibodies (mAbs) offer higher affinity and specificity for the target, hence a better benefit/risk profile, and long half-life - with advantages for dosing frequency^3,4^. However, mAbs may induce immune responses^5^, whose clinical effects vary widely: the formation of anti-drug antibodies can affect both safety (induction of hypersensitivity responses of different entity) and efficacy (neutralising the therapeutic antibodies or increasing their clearance)^6–8^.

Therapeutic drug monitoring (TDM), i.e. the measurement of drug concentrations in body fluids, is considered an essential tool to support clinicians in optimising dosage regimens and is routinely employed for some small molecules with narrow therapeutic windows and/or marked pharmacokinetic variability. TDM is increasingly proposed to guide the use of therapeutic antibodies, in view of the high inter-individual variability of their blood concentrations^9–14^. Parallel measurement of anti-drug antibodies (therapeutic drug and immunogenicity monitoring - TDIM))^15^ can help with objective decisions for the management of primary and secondary non-responders, or to avoid/reduce unnecessary interventions with these expensive treatments. The potential of TDIM for improving patients’ outcomes and for reducing costs is mainly suggested by studies with infliximab (IFX)^16,17^, approved by the Food and Drug Administration (FDA) in 1998.

IFX is a chimeric monoclonal IgG antibody against tumour necrosis factor α (TNFα), used to treat many chronic inflammatory conditions such as rheumatoid arthritis, spondylarthritis, Crohn’s disease, ulcerative colitis, psoriatic arthritis and psoriasis. More than 70% of patients experience primary or secondary loss of response (LOR)^18–22^ and in most instances treatment decisions after LOR are based on trial and error: higher doses of IFX are used at first to try to recover a clinical response, which may be successful in some patients whereas others are uselessly exposed to an expensive drug with side effects. Patients who continue to have LOR are usually switched to a different anti-TNFα antibody, assuming the presence of antibodies towards IFX (ATI), or they are switched to another class of agents. ATI were observed in different studies with variable incidence rates^11,23–26^; this variability possibly reflects differences in bioanalytical methods and interpretation approaches^15^.

The potential of TDIM is supported by studies showing that IFX trough levels (IFX-TL, i.e. the blood levels just before the next dose) correlate with clinical response^27–30^, with threshold concentrations of 3–7 μg/mL^10,13,31^. Algorithms have been proposed in which, for example, a LOR due to low IFX-TL without ATI suggests raising the dose or shortening the dosing interval, whereas in cases of low TL due to ATI it may be preferable to switch to another anti-TNFα^9,14,31^. The detection of significant levels of TNFα-binding IFX (i.e. active IFX) is sometimes considered sufficient to avoid the determination of ATI, though it has been proposed that ATI can have a deleterious effect on clinical outcome even when IFX levels are adequate^13^, and that ATI levels can guide clinical decision-making on intensifying treatment^32^. Clinical- and cost-effectiveness aspects of TDMI-based algorithms in comparison with the trial and error approach have also been claimed, according to randomised clinical trials^14,33^.

Several techniques have been used to measure IFX and ATI concentrations in sera, including solid phase enzyme-linked immunosorbent assays (ELISA)^23,24,27,34–38^, radioimmunoassays (RIA)^39–42^ and homogeneous mobility shift assays (HMSA)^10,13,43^. ELISA is the most common technique, mainly because it is relatively simple; RIA requires ^125^I-labeled reagents and laboratories equipped to use radioactive materials; HMSA requires labelling reagents with a fluorescent dye, and size-exclusion high-performance liquid chromatography apparatus for the analysis. All these approaches require a long incubation to reach binding equilibrium between sera IFX/ATI and detection targets, then washing, and steps for the detection of the bound complex, e.g. further incubation with secondary antibodies for ELISA. Multiple incubations and washing steps may affect the detection of low-affinity ATI^44^, and reduce the accuracy and precision of the measurements^35^.

The present study reports the characterisation and validation of surface plasmon resonance (SPR)-based assays to measure serum concentrations of IFX and ATI. SPR is widely used to study the interaction between two unlabelled molecules in real time, one immobilised on a sensor chip, the other flowing through a microfluidic system over the chip surface. SPR has significant advantages: TNFα and IFX are immobilised, as “capturing” molecules, on parallel surfaces of the *same* sensor chip, and the flow of patient’s sera on both of them results in *immediate* SPR signals, from which IFX and ATI concentrations are determined *simultaneously*, on the calibration curves.

We assessed the sensitivity and reproducibility of the SPR assays, as for the validation of any analytical method, and applied it to measure the TL of IFX and ATI in 15 patients treated for inflammatory bowel diseases (IBD), comparing the SPR results to those generated by a commercial ELISA kit.

## Materials and Methods

### Chemicals and reagents

Remicade® was from MSD Italia S.r.l. (Rome, Italy); CT-P13 (Inflectra) was from Hospira S.r.l. (Naples, Italy). 10x Dulbecco-PBS was obtained from Euroclone S.p.A. (Pero, Italy). MgCl_2_, ethylenediaminetetraacetate (EDTA) and Tween 20 were from Sigma-Aldrich (Milan, Italy). Water was provided in-house with a Milli-Q system (Millipore, Bedford, MA, USA). HCA-216 was purchased from Bio-Rad Laboratories (Segrate, Italy) and used as calibrator for ATI.

### Control serum

Blood was taken from healthy volunteers and collected into *VACUETTE®* tubes with Serum Clot Activator (ref. 456018, Greiner bio-one), then centrifuged. Serum samples were pooled, aliquoted and stored at −80 °C.

### Calibration curve and quality controls of IFX

Stock solutions of IFX (CT-P13 or Remicade, as indicated) were prepared in water at 10 mg/mL. The concentration was checked by measuring the absorbance at 280 nm using an extinction coefficient of 217440 M^−1^cm^−1^ ^45^. The stock solutions were diluted in water to prepare the working solutions and then used for calibration curves and quality controls (QC). Stock and working solutions were stored at −80 °C until use.

QC samples were generated by spiking aliquots of control serum with the appropriate amounts of IFX working solutions, to final concentrations of 0.5, 1, 3, 7 and 8 μg/mL. These QC samples were stored at −80 °C and assayed in different analytical sessions. For each analytical session an independent six-point calibration curve was generated with IFX in the range 0.5–8 μg/mL of serum.

### Calibration curve and quality controls of ATI

The stock solution of commercial ATI (HCA-216, 0.5 mg/mL) was stored at −80 °C until use. QC samples of ATI were prepared at final concentrations of 5, 11, 22.5, 34 and 40 μg/mL serum. These were stored at −80 °C and assayed in different analytical sessions. For each analytical session an independent six-point calibration curve was generated with ATI in the range 5–40 μg/mL serum.

### Patients

The study was approved by the Ethics Committees of the two participating hospitals (Humanitas Research Hospital Rozzano, Milan, Italy; and Careggi University Hospital, Florence, Italy). All patients provided informed consent. All procedures were in accordance with the ethical standards of the institutional and/or national research committee and with the 1964 Helsinki declaration and its later amendments, or comparable ethical standards.

Blood was taken from 15 patients treated with CT-P13 (Remsima®, Celltrion; Inflectra®, Pfizer) just before the infusion of a maintenance dose of CT-P13. The serum was immediately obtained from blood and stored at −80° until analysis. The main characteristics of the patients are summarised in Table S1.

### SPR assay

**Figure 1:**
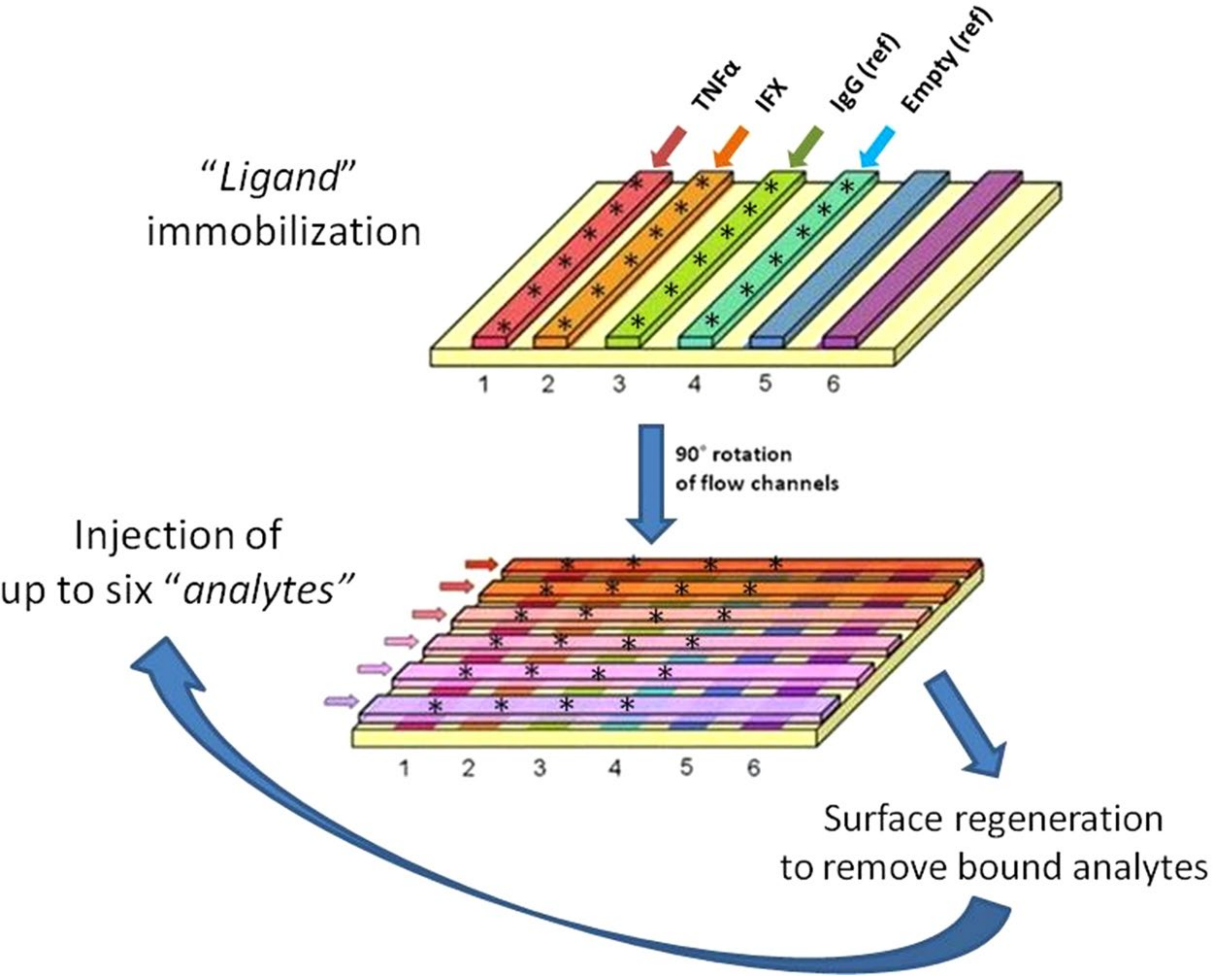
General scheme of the SPR-based assay for simultaneous determination of IFX and ATI concentrations in serum. The SPR apparatus was the ProteOn XPR36 Protein Interaction Array system (Bio-Rad), which has six flow channels which can immobilise up to six ligands on parallel strips of the same sensor surface (e.g. TNFα, IFX, suitable controls including IgG or an “empty” channel). The flow channels can be rotated 90° so that up to six analyte solutions can be flowed in parallel on all the immobilised ligands, creating a multi-spot interaction array (each spot indicated by *): for example, injection of six concentrations of the analyte standards gives interaction affinities or the calibration curves; and the injection of six serum samples can simultaneously evaluate their SPR-binding signals on TNFα, IFX and appropriate controls. The terms “ligands” and “analytes” are used throughout the manuscript as indicating the immobilised and the flowing interactants, respectively.

Figure 1 shows the main features of the proposed SPR-based assay. TNFα, IFX (Remicade or CT-P13, as indicated), and IgG (control) were immobilised using amine-coupling chemistry on the surface of a GLC sensor chip (BioRad), with a modified alginate-based polymer matrix bound to a gold surface. Briefly, the carboxyl groups on the matrix were activated with 0.04 mM sulfo-N-hydroxysuccinimide/0.3 mM 1-ethyl-3-(3-dimethylaminopropyl)-carbodiimide (sulfo-NHS/EDC) according to the manufacturer’s directions to form N-hydroxysuccinimide esters. The ligands were diluted in acetate buffer (pH 5.0) to give 5 μg/mL TNFα and 30 μg/mL IFX and IgG. These solutions were then flowed over the activated chip surface for 5 min at a rate of 30 μL/min, to ensure the spontaneous reaction of the ester with the primary amines on the protein to form covalent links. The remaining activated carboxyl groups were blocked with 1 M ethanolamine, pH 8.0. A reference “empty” surface was always prepared in parallel using the same immobilisation procedure but without the addition of a ligand.

After rotation of the fluidic system, analyte solutions were injected in parallel surfaces, so they flowed on all the immobilised ligands, creating a multi-spot interaction array (Fig. 1). Human sera containing IFX or ATI were subjected to acidic pre-treatment before injection. First, the samples were diluted 1:20 in 100 mM acetic acid pH 3 and incubated for 15 min at room temperature. Then they were diluted 1:1.5 in 0.5 M phosphate buffer pH 7.4 to a 30-fold overall dilution. The running buffer of the SPR instrument was 10 mM phosphate buffer containing 150 mM NaCl and 0.005% Tween 20 (PBST pH 7.4). Analytes flowed over immobilised ligands for 3 min at a rate of 30 μL/min. Dissociation was measured in the following 7–11 minutes. All these assays were done at 25 °C.

The sensorgrams (time course of the SPR signal in RU (1000 RU = 1 ng/mm^2^)) were normalised to a baseline of zero. The signals in the surfaces immobilising the ligands were corrected by subtracting the nonspecific response in the reference surface (“empty” surface for immobilised TNFα, and IgG for immobilised IFX). The kinetic parameters, dissociation rate constants (k_on_ and k_off_) and the equilibrium dissociation constant (K_D_) were obtained from globally fitting (nonlinear regression) the Langmuir model, implemented in ProteOn analysis software, to entire sensorgrams (association and dissociation phases) obtained by injecting different analyte concentrations.

The calibration curves for the method validation and analysis comprised six-point calibrators in the range of 0.25–8 μg/mL serum for IFX or 5–40 μg/mL serum for ATI. For each analytical session, two runs with calibrators were carried out, one at the beginning and one at the end of the session. Responses, expressed as the RU at the end of the dissociation phase, were plotted against the corresponding analyte concentration and the data were fitted using a weighted (1/x^2^) linear regression. All calibration curves analysed during method validation showed determination coefficients (r^2^) always over 0.99; the accuracy of the back-calculated concentrations was always within the acceptance limits (±15% of the nominal value).

The chip was regenerated by injecting a 3.3 M MgCl_2_ solution containing 5 mM EDTA.

### ELISA

For the ELISA assays we used the Lisa-Tracker Duo Infliximab (Theradiag) according to the manufacturer’s instructions.

## Results

### Method characterisation

The capturing ligands (TNFα or IFX) and IgG (as reference) were covalently immobilised on parallel lanes of the same sensor chip (Fig. 1); one lane was kept “empty”, i.e. without any immobilised ligand, as an additional reference. Each lane included six interaction spots, to allow for the simultaneous examination of six different analytes flowed in the subsequent steps (Fig. 1). The amounts of TNFα, IFX and IgG immobilised on each interaction spot were about 1500, 6000 and 6500 RU, respectively. The variability (SD) on the six spots of the same lane was always <10% (Fig. 1).

The method was characterised by spiking IFX or commercial ATI in serum from healthy subjects, to clinically relevant concentrations. Before injection in the SPR apparatus, sera were diluted 1:30 in PBST to reduce the bulk effects (broken red line in Fig. S1a).

Fig. S1a,b shows representative raw sensorgrams obtained injecting the serum - with or without IFX or ATI - on immobilised ligands (TNFα or IFX) or reference surfaces. Apart from the serum-dependent bulk effect, common to all the combinations, the main signals detectable at the end of the dissociation phase were for IFX-spiked serum on immobilised TNFα (Fig. S1a) and ATI-spiked serum on immobilised IFX (Fig. S1b). The *specific* binding of IFX to TNFα could be precisely determined by subtracting the signal measured in parallel on the reference “empty” surface (Fig. S1c); similarly the *specific* binding of ATI to immobilised IFX was obtained by subtracting the signal measured in parallel on the surface coated with IgG (Fig. S1d).

**Figure 2:**
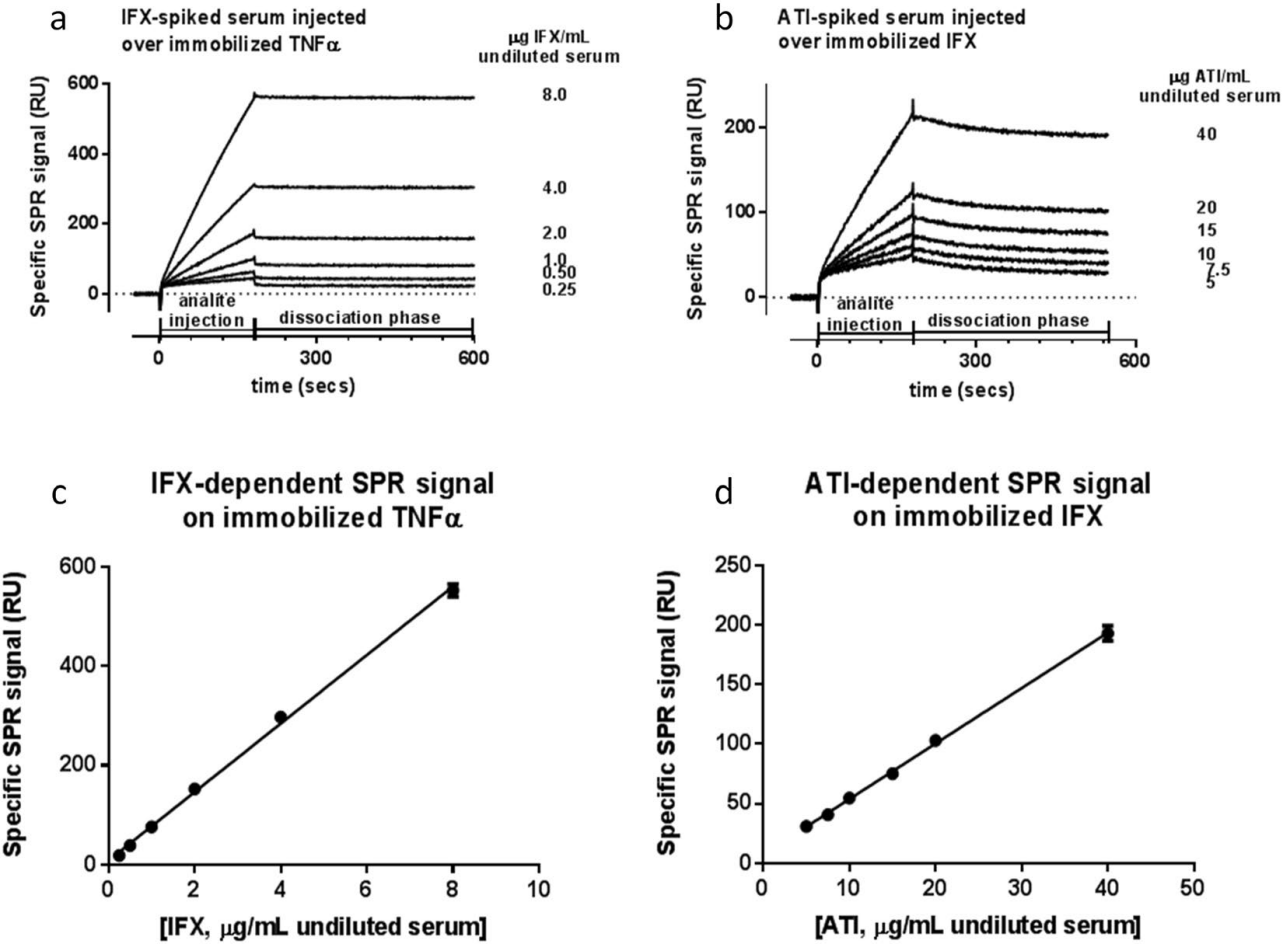
Linearity between specific SPR signal and serum concentrations of IFX (A) or ATI (B). Serum from healthy volunteers was spiked with IFX (CT-P13) or ATI to the indicated concentrations then diluted 30-fold before injection into the SPR instrument. (**a**) Representative sensorgrams obtained injecting simultaneously the six dilutions of IFX-spiked sera over immobilised TNFα, after subtraction of the SPR signals from the parallel empty surfaces (see Fig. 1) for the experimental design and Fig. S1 for raw data). (**b**) Representative sensorgrams obtained injecting simultaneously the six dilutions of ATI-spiked sera over immobilised IFX, after subtraction of the SPR signals from the parallel surfaces coated with IgG. (**c**) Linearity between IFX concentration and specific SPR signal (at the end of the dissociation phase). Points show the mean ± SD for six consecutive injections, each as in panel a. The mean r^2^ of the six straight lines is 0.997 ± 0.001; the equation obtained from the linear regression is: y = 69.01(±1.37) x + 8.36(±5.16). (**d**) Linearity between ATI concentration and specific SPR signal (at the end of the dissociation phase). Points show the mean ± SD for six consecutive injections, each as in panel b. The mean r^2^ of the six straight lines is 0.998 ± 0.002; the equation obtained from the linear regression is: y = 4.65(±0.07) x + 7.30(±1.34).

Figure 2 shows representative sensorgrams of the specific binding signals with different concentrations of IFX (Fig. 2a) or ATI (Fig. 2b). Fitting these sensorgrams indicated very high affinities, with apparent K_D_ in the low pM range, mainly due to very slow dissociation, suggesting avidity effects (one bivalent antibody binding to two immobilised molecules). No significant differences were observed on injecting serum spiked with Remicade or CT-P13 on TNFα, or injecting ATI-spiked serum over immobilised Remicade or CT-P13 (data not shown).

The very slow dissociation made it essential to identify a suitable “regeneration procedure”, capable of rapidly and completely removing the bound antibody without affecting the binding capacity of the immobilised ligand. We tested many different “regeneration solutions”^46^ (data not shown) and eventually determined that a MgCl_2_/EDTA solution completely removed the analytes, without altering the binding properties of the sensor surface even after numerous cycles (Fig. S2**)**.

### Analytical performance

Repeated analysis reliably confirmed that specific SPR signals correlated linearly with the serum concentrations of spiked IFX (Fig. 2c) or ATI (Fig. 2d), with coefficient of determination (r^2^) of the linear regression always >0.99. The lower limits of quantification, defined as 10*SD_blank_ (20 RU), were 0.20 μg/mL for IFX and 2.5 μg/mL for ATI.

We then examined whether the method can measure − accurately and reproducibly − the concentrations of IFX and ATI in QC samples. Aliquots of these solutions were stored at −80 °C and assayed in different analytical sessions. IFX and ATI concentrations were calculated by reference to a calibration curve as in Fig. 2c,d, prepared *ex-novo* and independently from the QCs. Accuracy was determined by expressing the calculated concentration as a percentage of the nominal concentration and, according to EMA guidelines, has to be within 15% of the nominal value for each concentration (±20% for the LLOQ as an exception). Precision, expressed by the CV (%), must not exceed 15% for all concentrations (20% for the LLOQ). Tables S2–5 show that the intra-day and inter-day accuracy and precision were within the required limits. These data also indicated that IFX and ATI are stable for up to one month, when stored at −80 °C in serum.

We also checked for matrix effects, spiking different concentrations of IFX or ATI in the serum from six different subjects. SPR indicated very low inter-subject variability (% CV was always less than 10%, data not shown), thus excluding appreciable matrix effects. It should also be considered that circulating TNFα concentrations (up to 100 pg/mL in pathological conditions) are much lower than the therapeutic concentrations of IFX (relevant TL > 100 ng/mL), excluding effects on the determination of the antibody concentration.

**Figure 3:**
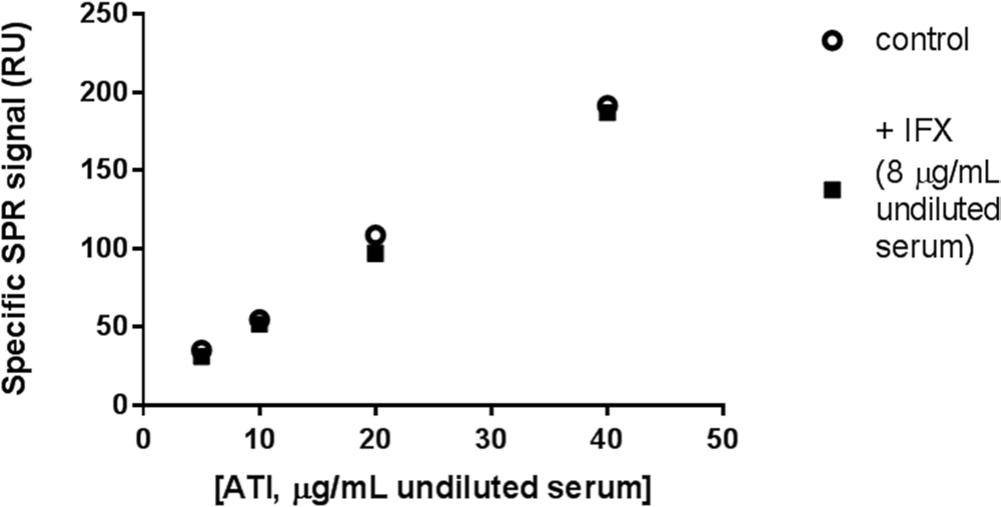
Effect of IFX on determination of ATI concentration. Aliquots of human serum from healthy volunteers were spiked with commercial ATI, to the final concentrations indicated, and with IFX (CT-P13, final concentration 8 μg/mL) or its vehicle. Serum aliquots were diluted 30-fold, subjected to acidic pretreatment, and injected over immobilised IFX and IgG (the latter for evaluation of non-specific binding). The points indicate the specific SPR signal due to the binding of flowing ATI to immobilised IFX.

We then evaluated the cross-interference of IFX on the determination of ATI concentrations. This is a well-known limitation of ELISA that often prevents accurate measurement of ATI in the presence of IFX, although an acidic dissociation step was proposed to reduce this problem. In our SPR assay, which includes acidic pre-treatment, we did not find any interfering effect at the highest concentration of IFX (8 μg/mL undiluted serum) even at the lowest ATI concentration of 5 μg/mL undiluted serum (Fig. 3).

### Analysis of serum of patients treated with IFX and comparison with ELISA

We applied the SPR to analyse serum samples from 15 patients treated with IFX for inflammatory bowel disease (either Crohn’s disease or ulcerative colitis) (Table S1). Blood samples were taken just before a maintenance infusion, in order to measure the trough levels of IFX, and the presence of ATI. The results were compared with the results of analysing the same serum samples with the commercial LISATRACKER Duo Infliximab (Theradiag) according to the manufacturer’s directions (Table 1**)**. There was a very good correspondence between the serum IFX concentrations measured with ELISA and the SPR assay. The Pearson correlation coefficient, determined on the 10 sera with measurable IFX levels, was 0.9912 (p < 0.001).

**Table 1:** Correlation between infliximab (IFX), anti-IFX antibodies (antibodies towards IFX, ATI) determined by SPR and ELISA in the serum of patients treated with IFX (trough levels, just before the next infusion) and clinical characteristics of the patient population during the study period.

PT ID	Centre	Diagnosis	Months of IFX therapy	Dose regimen at sampling	Disease activity	Concomitant thiopurines	IFX (μg/mL undiluted serum)		ATI (μg Eq/mL undiluted serum)^§^	
							SPR	ELISA	SPR	ELISA
#1	ICH	CD	29	2 = 5 mg/kg E4W	Remission	No	4.76 ± 0.03	3.89–4.55	<LLOQ	<LLOQ
#2	ICH	CD	21	5 mg/kg E8W	Moderate	No	7.70 ± 0.13	6.92–7.86	<LLOQ	<LLOQ
#3	ICH	CD	29	5 mg/kg E8W	Remission	No	3.03 ± 0.03	3.57–3.22	<LLOQ	<LLOQ
#4	ICH	UC	97	5 mg/kg E8W	Remission	No	2.31 ± 0.01	2.57–2.74	<LLOQ	<LLOQ
#5	ICH	CD	46	5 mg/kg E8W	Remission	No	2.63 ± 0.02	2.76-2.77	<LLOQ	<LLOQ
#6	ICH	CD	30	5 mg/kg E8W	Mild	No	<LLOQ	<LLOQ	37.57 ± 0.21	3.9-4.0*
#7	ICH	CD	22	5 mg/kg E8W	Remission	No	<LLOQ	<LLOQ	<LLOQ	0.04–0.07
#8	ICH	UC	7	5 mg/kg E8W	Remission	Yes	<LLOQ	<LLOQ	6.64 ± 0.14	*over*
#9	ICH	CD	30	5 mg/kg E8W	Remission	No	7.48 ± 0.02	6.61–7.90	<LLOQ	<LLOQ
#10	ICH	UC	3	5 mg/kg E8W	Remission	No	<LLOQ	<LLOQ	5.67 ± 0.10	0.57–0.63*
#11	ICH	CD	7	5 mg/kg E8W	Remission	No	1.39 ± 0.05	1.52–1.69	2.54 ± 0.04	<LLOQ
#12	FI	CD	4	5 mg/kg E8W	Mild	Yes	4.62 ± 0.10	4.84–5.32	<LLOQ	<LLOQ
#13	FI	CD	4	5 mg/kg E8W	Remission	No	4.09 ± 0.03	4.13–4.32	<LLOQ	<LLOQ
#14	FI	CD	5	5 mg/kg E8W	Remission	No	4.11 ± 0.04	3.98–4.35	<LLOQ	<LLOQ
#15	FI	UC	7	5 mg/kg E8W	Remission	No	<LLOQ	<LLOQ	3.60 ± 0.34	0.08-0.09

For SPR data, each value is the mean ± SD of three independent replications; the single values of the two replications are shown for ELISA. ^§^ATI are expressed as μg Equivalents/mL, to highlight that the ATI used for the calibration curves are different from those produced by the patients. ICH: Humanitas Research Hospital (Rozzano, Milan, Italy); FI: Careggi University Hospital (Florence, Italy) Lower limits of quantifications (LLOQ) for SPR analysis were 0.2 μg/mL for IFX and 2.5 μg Eq/mL for ATI. LLOQ for ELISA was set at the lowest point of the calibration curve: 0.5 μg/mL for IFX and 0.025 μg Eq/mL for ATI. *Extrapolated, since the highest point of the calibration curve was 0.2 μg Eq/mL for ATI. “Over” indicates a value not measurable in the spectrophotometer.

Results were more complex for ATI. ELISA detected ATI in serum samples from five patients: two had very high levels, out of scale (≥4 µg Eq/mL serum) and three had 0.04–0.63 µg Eq/mL serum. All but one of these ATI-positive serum samples were ATI-positive with SPR as well; SPR also detected ATI in a serum considered ATI-negative by ELISA (patient #11).

Despite the overall qualitative correspondence between the two assays, absolute concentrations differed by one or two orders of magnitude (Table 1); theoretically, the low ATI levels detected by ELISA in patients #10 and #15 should have not been detectable by SPR (LLOQ 2.5 μg/mL serum). We can exclude that this was due to artefacts related to the different ATI used for the calibration curve since the SPR signals were superimposable by injecting similar concentrations of the two ATI (Fig. S3). However, the ATI used for the calibration curve had a much slower dissociation rate constant than the patient’s ATI (Fig. S4), and these constants served to estimate the amount of ATI dissociating from IFX during the second incubation step in the ELISA (Fig. S4). These data support the possibility that a patient’s ATI may significantly dissociate from IFX during this ELISA incubation step while the ATI used for the calibration does not. This results in a significant underestimation of the actual concentration of the former.

This problem has less impact in SPR studies, which monitor binding events in real time without long incubation steps. This quite likely explains why the SPR assay detected ATI in the serum of patient #11 which appeared ATI-negative with ELISA; this patient’s ATI had a fast dissociation rate constant, e.g. faster than that of patient #15 (Fig. S4). The possibility of monitoring and comparing the ATI’s dissociation rate constants is a further added value of the SPR assay.

ATI were only found in IFX-negative sera, with the notable exception of patient #11 (Table 1). We considered it unlikely that the lack of ATI in IFX-positive sera was just a technical artefact due to masking effects of IFX, since SPR studies were done after acidic pre-treatment, which proved highly effective in preliminary characterisation (Fig. 3).

## Discussion

The proposed SPR-based approach gives reliable and convenient measurements of serum concentrations of IFX and ATI, supporting its application for TDIM. In comparison to ELISA and the other techniques proposed so far (RIA^39^, HMSA^43^, electrochemiluminescence assays^47^) SPR has obvious advantages in that it does not require labelled compounds and avoids long incubation/separation/detection steps, reducing the complexity and associated variability. Some reports have already appeared where SPR was used to measure IFX^48,49^ or anti-drug antibodies^50–52^, but this is the first study addressing the simultaneous analysis of both. Most importantly, we demonstrate the advantages of SPR through rigorous characterisation and validation of the assay performances, to fulfil the requirements for reliable analysis of clinical samples. Similar method validation is often lacking for other techniques.

The highly efficient procedure for surface regeneration allows dozens of consecutive injections on the same chip, significantly reducing the cost of these analyses that become competitive with ELISA. A cycle of injection of serum samples and chip regeneration takes approximately 20 min, with simultaneous measurement of IFX and ATI.

Serum concentrations of free (active) IFX were determined from the specific SPR binding signal on immobilised TNFα. The reliability of these values is based on the linearity of the calibration curve in a clinically relevant range (0.5–8 μg/mL undiluted serum) and on the high accuracy and precision of the method. No specific binding signal was observed with serum from healthy volunteers, indicating that soluble TNF receptors (sTNFR1 and sTNFR2) were not detectable under these experimental conditions. Although higher blood levels of sTNFRs have been found in patients with inflammatory bowel diseases or rheumatic autoimmune diseases^53,54^, the size of these increases (always less than two-fold) makes any important interaction with the assay unlikely. The trough IFX levels measured by SPR in 15 patients were superimposable with those given by a commercial ELISA, which also detects free IFX by its binding to immobilized TNFα.

Serum concentrations of ATI were determined from the specific SPR signal on immobilised IFX. The method was validated with control serum spiked with a commercial ATI, and showed high accuracy and precision; calibration curves were linear in the range of 5–40 μg/mL undiluted serum with an estimated LLOQ of 2.5 μg/mL. These concentrations were markedly higher than those detectable with ELISA (25–200 ng/mL serum), and the difference can be explained by the need to dilute serum for SPR assay (30-fold vs 2-fold for ELISA) and the signal amplification intrinsic to ELISA. However, SPR detected ATI in all but one patient’s sera where ATI were detected by ELISA, even when the levels with ELISA should not have been sufficient for SPR detection. This apparent discrepancy is due to an underestimation of ATI levels using ELISA because the ATI used for the calibration curve have a much higher affinity for IFX than the patient’s ATI - in particular a much slower dissociation rate constant. In fact, high-affinity ATI do not dissociate during the long incubation with the secondary antibody (after the unbound ATI have been washed away) whereas lower-affinity ATI may dissociate: thus a low concentration of the high-affinity ATI (the one used for the calibration curve) will produce the same ELISA signal as a high concentration of a low-affinity ATI (the patient’s ATI), resulting in significant underestimation of the concentration of the latter. This is consistent with previous data showing that the limit of detection of ELISA is inversely proportional to the affinity of the tested ATI^50^.

Underestimation of ATI values with the ELISA kit used here (Lisa-Tracker) was also reported in comparison with another home-made ELISA test^35^ and this might be due to the different ATI used for the calibration curves. A calibrator^16^ can only be used to compare the results from different laboratories, and cannot avoid errors in the quantification of a patient’s ATI if they have different binding properties. In general terms this is true for any analytical assay, but ELISA seems particularly sensitive because of its long incubation steps where binding dynamics markedly affect the final output, with underestimation of the real concentrations by up to 2–3 orders of magnitude (these and published^50^ data).

The possibility that ELISA may fail to detect low-affinity antibodies has already been indicated^44^. Since SPR measures the binding events in a much shorter time, its results can be expected to be much less affected. SPR detected ATI in a serum (patient #11) which appeared ATI-negative with ELISA, and showed that these ATI had the fastest dissociation rate from IFX. Interestingly, this was the only ATI-positive serum in which IFX was also detected.

Apart from this patient, and the quantitative differences between SPR and ELISA, mostly associated with ELISA’s intrinsic shortcomings, both methods detected ATI only in the five IFX-negative serum samples. We consider it unlikely that the lack of ATI in the other sera was just a technical artefact due to masking effects of IFX, since we verified that high concentrations of IFX had no effect on ATI detection, even with the lowest ATI concentration. The tolerance to SPR-based detection of ATI, after acidic pre-treatment (as in our conditions), has already been described^51^ and may be because only one arm of the ATI is free for detection. Moreover, the possibility of re-association between ATI and IFX, when physiological pH is re-established after the acidic pre-treatment, is more likely during the long incubation step of ELISA (at least one hour) than during the short SPR injection step (few minutes).

According to our TDIM analysis, four groups could be identified in the 15 patients: (i) eight patients (53%) were in remission and had IFX levels in the proposed therapeutic range (3–8 μg/mL)^14^ and no ATI; (ii) three patients (20%) were in remission although IFX levels were below the cut-off and ATI levels were measurable;these patients may thus benefit from stopping treatment, to avoid potential toxic effects; (iii) two patients (13%) with active disease had ATI and no measurable IFX; these patients may benefit from switching to a different anti-TNFα antibody. (iv) two patients (13%) had active disease despite adequate IFX levels and no ATI: these patients may benefit from switching to another class of agents.

Despite the very small number of patients, these data confirm wide inter-individual variability^9–14^, and strongly uphold the usefulness of TDIM. It is important to recall that high interindividual variability has been described for the reference IFX Remicade^10,13,27,55^, very similar to that seen with CT-P13 after switching^56^.

In summary, systematic characterisation and validation of a novel SPR-based analytical assay showed that it provides highly reliable measurements of serum concentrations of IFX and ATI, which are useful for TDIM. The specific features of SPR, mainly the possibility of *directly* detecting and measuring serum antibodies in a very short experimental time (a few minutes), offer further significant advantages over classic ELISA, particularly the more reliable measurement of low-affinity patient’s ATI. The assay allows for much faster analysis, partly also because of the simultaneous measurement of IFX and ATI from the same serum sample; it is possible to regenerate the sensor and run dozens of analytical sessions on the same chip, resulting in cost-effectiveness over commercial ELISA kits. Similar SPR-based analytical assays can be tested and developed for other biotherapeutics. In addition, point-of-care devices based on SPR technology could be developed for very rapid on-site analysis^48,57^, boosting the flexibility of TDIM.

## Supplementary information

Supplementary material

## Data Availability

The datasets generated during and/or analysed during the current study are available from the corresponding author on reasonable request.
