# Supplementary material for: A Surface Plasmon Resonance-based assay to measure serum concentrations of therapeutic antibodies and anti-drug antibodies
Source: Sci Rep. 2019 Feb 14;9:2064. doi: 10.1038/s41598-018-37950-4 (PMC6376047; doi:10.1038/s41598-018-37950-4)
Supplement: Supplementary file 1 — Supplementary material [file 41598_2018_37950_MOESM1_ESM.pdf]

Supplementary material

A Surface Plasmon Resonance-based assay to measure serum concentrations of therapeutic antibodies and anti-drug antibodies

Marten Beeg[1], Alessandro Nobili[1], Barbara Orsini[2], Francesca Rogai[2], Daniela Gilardi[3], Gionata Fiorino[3,4], Silvio Danese[3,4], Mario Salmona[1], Silvio Garattini[1], Marco Gobbi[1]

Affiliations

1) Istituto di Ricerche Farmacologiche Mario Negri IRCCS, Milan, Italy
2) Clinical Gastroenterology Unit, Careggi University Hospital, Florence, Italy
3) IBD Center, Dept. of Gastroenterology, Humanitas Clinical and Research Institute, Rozzano, Milan, Italy
4) Department of Biomedical Sciences, Humanitas University, Rozzano, Milan, Italy

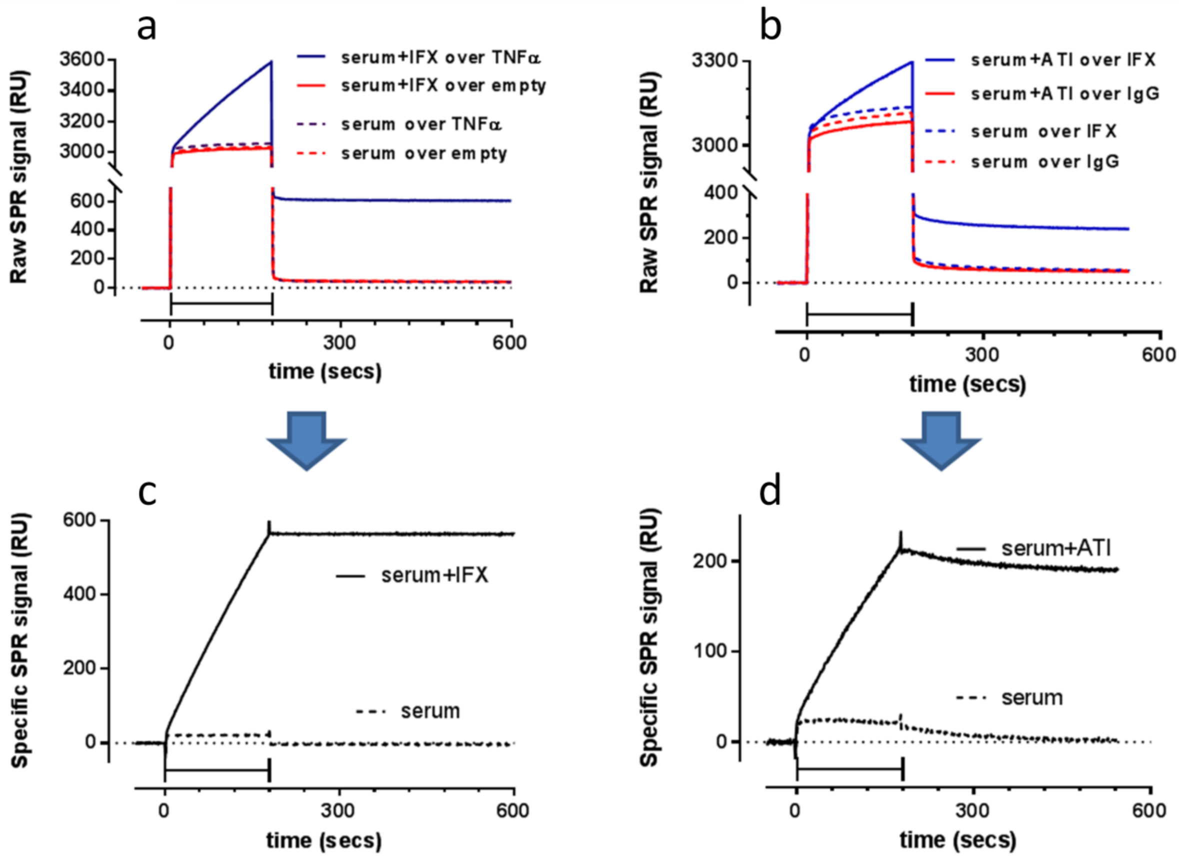

Fig S1: Characterisation of the SPR assay for specific recognition of IFX-TNFa and ATI-IFX interactions.

Panel a shows the raw sensorgrams obtained injecting diluted serum, with or without IFX, over two parallel sensor surfaces, one immobilising TNFα and the other left empty for reference (see also Fig. 1 for details of the experimental design). In this example, a control human serum was mixed with 8 μg/mL IFX (CT-P13) or vehicle, diluted 30 times and injected for 3 min as indicated; dissociation was then followed for 7 min. Panel c reports the sensorgrams obtained from A after subtraction of the signal in the empty surface (i.e. blue sensorgrams minus the corresponding red ones), showing the specific binding to immobilised TNFα.

Panel b shows the raw sensorgrams obtained injecting diluted serum, with or without ATI, over two parallel sensor surfaces, one immobilising IFX and the other immobilising IgG. In this case, the control human serum was mixed with 40 μg/mL ATI, diluted 30 times and injected for 3 min as indicated; dissociation was then followed for 6 min. Panel d reports the sensorgrams obtained from B after subtraction of the signal in the surface coated with IgG (i.e. blue sensorgrams minus the corresponding red ones), thus showing the specific binding to immobilized IFX.

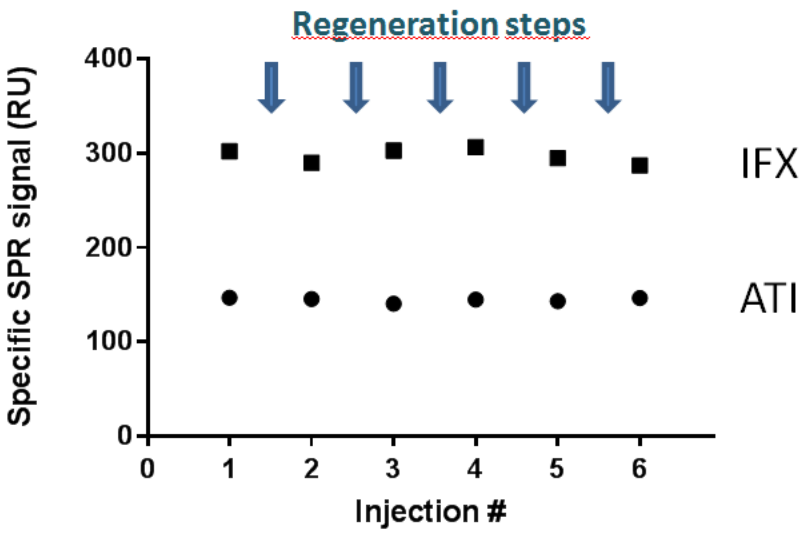

Fig S2: Efficacy of chip regeneration after analyte injections.

Serum from healthy volunteers was spiked with 4 µg/mL IFX (CT-P13) or 20 µg/mL ATI diluted 30-fold and injected into the SPR instrument in six consecutive sessions, with regeneration steps in between.
Squares indicate the specific binding of IFX on immobilized TNFα, and circles indicate the specific binding of ATI on immobilized IFX (specific binding signals were obtained as described in Fig S1).

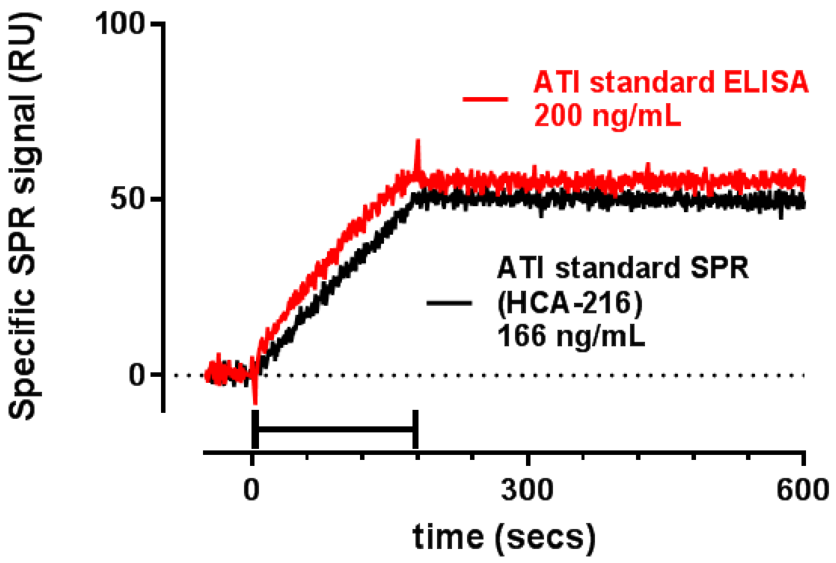

Fig S3: Specific SPR binding signal of the two ATI calibrators for immobilised IFX.

Sensorgrams obtained injecting HCA-216 (the ATI used as calibrator in the SPR assays) and the ATI used as calibrator in the ELISA kit (Lisa Tracker, Theradiag) over IFX immobilised on the sensor chip. In this case, for comparison, the concentrations are those actually injected. Sensorgrams show specific signals obtained after subtraction of the SPR signals from in the parallel surface coated with IgG (see Fig S1B-D) and after subtraction of the corresponding vehicle (buffer for ELISA-ATI and serum 1:30 for SPR-ATI). This latter normalisation permits a proper comparison of the association and dissociation rates of the specific ATI-IFX interaction without confounding factors.

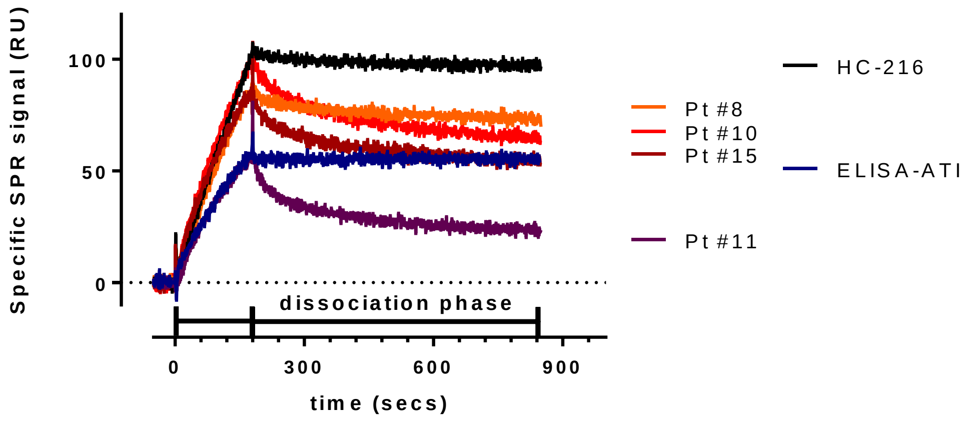

ATI	k_{off} (s^{-1})	expected dissociation in 60 min (%)
ELISA-ATI	$<1x10^{-6}$	100
HCA-216	$4.0x10^{-5}$	87
Pt #6	$7.3x10^{-5}$	77
Pt #8	$1.1x10^{-4}$	67
Pt #10	$3.5x10^{-4}$	28
Pt #11	$6.5x10^{-4}$	10
Pt #15	$3.0x10^{-4}$	34

Fig S4: Kinetics of specific binding of calibrators ATI and patient's ATI to immobilised IFX

The upper panel compares of the time-course of the specific SPR binding signals (sensorgrams) obtained with patient's diluted serum or calibrators ATI (HCA-216 used for calibration curves in SPR assays and the ATI used as calibrator in the ELISA kit). Sensorgrams show specific signals, obtained after subtraction of the SPR signals from in the parallel surface coated with IgG (see Fig S1B-D). Note that calibrators ATI dissociate from immobilised IFX at a much slower rate than patient's ATI. Dissociation rate constants were measured and are indicated in the table (also including Pt #6, not shown in the figure for graphical purposes). These koff enable one to calculate the percentage of bound ATI that dissociate after 60 min, i.e. the length of the second incubation step in the ELISA assay (see also the text).

Table S1. Clinical characteristics of the patient population at inclusion

**Diagnosis**	CD	11 (73%)
	UC	4 (27%)
**Age at inclusion (years)**	Median	39 (22-65)
**IFX therapy duration (months)**	Median	22.7 (3-97)
**Dose regimen**	5 mg/kg Q8	14 (93.3%)
	5 mg/kg Q4	1 (6.7%)
**Disease activity**	Remission	12 (80%)
	Mild	2 (13.3%)
	Moderate	1 (6.7%)
**Previous optimisation**	No	15 (100%)
	Yes	0
**Combination therapy with thiopurines**	Previous	2 (13.3%)
	Current	2 (13.3%)
	No	11 (73.4%)
**Concomitant steroids**	No	15 (100%)
	Yes	0
CD - Crohn's disease; UC - ulcerative colitis; IFX - infliximab; Q8 - every 8 weeks; Q4: every 4 weeks		

Table S2: Intra-day accuracy and precision for SPR analysis of IFX concentrations in human serum.

QC nominal concentration (μg/mL)	Calculated concentration (μg/mL)						Mean (μg/mL)	SD	Accuracy (%)	Precision (%)
	Run 1	*Run 2*	*Run 3*	*Run 4*	*Run 5*	*Run 6*				
**0.5**	0.45	0.34	0.41	0.47	0.46	0.42	0.42	0.048	84.92	11.27
**1**	0.99	0.88	0.97	0.97	1.01	0.97	0.96	0.045	96.38	4.70
**3**	3.19	2.85	2.33	2.88	2.87	2.94	2.84	0.279	94.78	9.81
**7**	6.69	6.88	6.87	6.95	6.79	6.91	6.85	0.094	97.80	1.37
**8**	7.41	7.44	7.44	7.38	7.26	7.48	7.40	0.078	92.49	1.06

Six SPR runs were carried out consecutively the same day, each including five QCs at the indicated nominal concentrations of spiked IFX (in μg/mL undiluted control serum). Two additional runs, one before and one after the six runs with QCs, were used for injection of the IFX calibrators prepared ex-novo and independently from the QCs. QC concentrations were calculated by interpolation from the calibration curve (example in Fig 2C). Accuracy was determined by expressing the mean calculated concentration as a percentage of the nominal concentration and had to be within 15% of the nominal value. Precision, expressed by the SD (%), had not to exceed 15%.

Table S3: Inter-day accuracy and precision for SPR analysis of IFX concentrations in human serum by SPR.

Nominal concentration (μg/mL)	Day 1		Day 2		Day 7		Day 30		Mean (μg/mL)	SD	Accuracy (%)	Precision (%)
**0.5**	0.47	0.34	0.38	0.40	0.43	0.54	0.47	0.48	0.44	0.064	87.93	14.65
**1**	0.84	1.18	1.06	1.00	1.30	1.08	0.97	0.97	1.05	0.143	104.9	13.63
**3**	3.20	3.13	2.91	2.95	2.79	3.02	3.01	3.12	3.02	0.133	100.54	4.42
**7**	6.43	7.84	7.63	7.30	7.36	7.10	6.86	6.73	7.16	0.471	102.22	6.58
**8**	7.63	7.43	7.97	7.94	7.68	7.68	7.76	7.72	7.73	0.172	96.57	2.23

Different SPR runs were carried out different days, in duplicate, each including five QCs at the indicated nominal concentrations of spiked IFX (inμg/mL undiluted control serum). IFX calibrators (six concentrations, 0.5-8 μg/mL undiluted serum) prepared ex-novo and independently from the QC, were also injected every day. QC concentrations were calculated by interpolation from the calibration curves (example in Fig 2C). Accuracy was determined by expressing the mean calculated concentration as a percentage of the nominal concentration and had to be within 15% of the nominal value. Precision, expressed by the SD (%), had not to exceed 15%.

Table S4: Intra-day accuracy and precision for SPR analysis of ATI concentrations in human serum.

QC nominal concentration (µg/ml)	Calculated concentration (µg/ml)						Mean (µg/ml)	SD	Accuracy (%)	Precision (%)
	Run 1	*Run 2*	*Run 3*	*Run 4*	*Run 5*	*Run 6*				
**5**	5.57	5.53	5.43	6.49	5.71	5.52	5.82	0.38	114.12	6.50
**11**	11.20	11.12	10.76	11.72	11.70	11.47	11.33	0.37	102.99	3.29
**22.5**	22.30	22.24	21.99	22.48	22.40	22.16	22.26	0.18	98.95	0.79
**34**	33.13	33.16	32.94	32.90	32.24	32.65	32.84	0.35	96.58	1.06
**40**	38.83	37.82	38.00	38.61	36.99	38.26	38.09	0.65	95.21	1.72

Six SPR runs were carried out consecutively the same day, each including five QCs at the indicated nominal concentrations of spiked ATI (in µg/mL undiluted control serum). Two additional runs, one before and one after the six runs with QCs, were used for the injection of the ATI calibrators prepared ex-novo and independently from the QCs. QC concentrations were calculated by interpolation from the calibration curve (example in Fig 2D). Accuracy was determined by expressing the mean calculated concentration as a percentage of the nominal concentration and had to be within 15% of the nominal value. Precision, expressed by the SD (%), had not to exceed 15%.

Table S5: Inter-day accuracy and precision for SPR analysis of ATI concentrations in human serum by SPR.

Nominal concentration (µg/mL)	Day 1		Day 2		1 Week		1 Month		Media (µg/mL)	SD	Accuracy (%)	Precision (%)
**5**	4.29	4.39	4.24	4.54	4.45	4.48	4.48	4.48	4.42	0.105	88.36	2.38
**11**	9.94	10.08	9.90	10.24	11.14	10.44	10.63	9.99	10.30	0.426	93.60	4.14
**22.5**	22.11	22.12	20.86	20.73	21.11	21.39	20.95	21.23	21.31	0.537	94.71	2.52
**34**	32.79	33.44	31.82	32.25	34.09	33.66	32.38	33.15	32.95	0.777	96.91	2.36
**40**	39.66	39.84	38.46	38.79	39.92	38.94	38.36	38.99	39.12	0.611	97.80	1.56

Different SPR runs were carried out different days, in duplicate, each including five QCs at the indicated nominal concentrations of spiked ATI (in µg/mL undiluted control serum). ATI calibrators (six concentrations, 5-40 µg/mL undiluted serum) prepared ex-novo and independently from the QC, were also injected every day. QC concentrations were calculated by interpolation from the calibration curves (example in Fig 2D). Accuracy was determined by expressing the mean calculated concentration as a percentage of the nominal concentration and had to be within 15% of the nominal value. Precision, expressed by the SD (%), had not to exceed 15%.
